# The potential of visible blue light (405 nm) as a novel decontamination strategy for carbapenemase-producing enterobacteriaceae (CPE)

**DOI:** 10.1186/s13756-019-0470-1

**Published:** 2019-01-17

**Authors:** Fenella D. Halstead, Zahra Ahmed, Jonathan R. B. Bishop, Beryl A. Oppenheim

**Affiliations:** 1 NIHR Surgical Reconstruction and Microbiology Research Centre, Queen Elizabeth Hospital, B15 2GW, Birmingham, UK; 2 Queen Elizabeth Hospital, University Hospitals Birmingham NHS Foundation Trust, B15 2GW, Birmingham, UK

**Keywords:** Blue light, Decontamination, Biofilm, CPE

## Abstract

**Background:**

Carbapenemase-producing *Enterobacteriaceae* (CPE) pose a considerable threat to modern medicine. New treatment options and methods to limit spread need to be investigated. Blue light (BL) is intrinsically antimicrobial, and we have previously demonstrated significant antimicrobial effects on biofilms of a panel of isolates, including two CPEs.

This study was performed to assess the antibacterial activity of 405 nm BL against a panel of CPE isolates (four encoding *bla*_NDM_, three *bla*_KPC_, two *bla*_OXA-48_, and three encoding both NDM and OXA-48 carbapenemases).

**Methods:**

In vitro experiments were conducted on 72 h old biofilms of CPEs which were exposed to 60 mW/cm^2^ of BL. Changes to biofilm seeding were assessed by measuring the optical density of treated and untreated biofilms.

**Results:**

Twelve bacterial clinical isolates (comprising eight *Klebsiella pnemoniae*, one *K. oxytoca*, and three *Escherichia coli*) were tested. BL was delivered for 5, 15 and 30 min, achieving doses of 162, 54, and 108 J/cm^2^, respectively.

All of the CPEs were susceptible to BL treatment, with increasing reductions in seeding with increasing durations of exposure. At 30 min, reductions in biofilm seeding of ≥80% were observed for 11 of the 12 isolates, compared to five of 12 after 15 min. CPE_8180 was less susceptible than the rest, with a maximum reduction in seeding of 66% at 30 min.

**Conclusions:**

BL is effective at reducing the seeding of mature CPE biofilms in vitro, and offers great promise as a topical decontamination/treatment agent for both clinical and environmental applications.

**Electronic supplementary material:**

The online version of this article (10.1186/s13756-019-0470-1) contains supplementary material, which is available to authorized users.

## Background

Infections caused by multidrug-resistant organisms and other nosocomial pathogens are associated with increased morbidity and mortality, and are estimated to cost the UK National Health Service >£1 billion a year [1]. *Enterobacteriaceae* are a common cause of community- and hospital-acquired infections, and are becoming increasingly multiresistant to first- and second-line antibiotics [2]. Carbapenemase-producing *Enterobacteriaceae* (CPE) are a particular concern, and have evolved due to the *Enterobacteriacae* group acquiring transferable β-lactamases which have recently evolved to confer resistance to the carbapenem (as well as penicillin and cephalosporin) classes of antibiotics. Treatment options for CPEs are severely restricted, and hence the rapid global increase and spread of CPEs has become a public health crisis, threatening delivery of healthcare and patient safety [3].

The UK reported a large increase in the number of CPE isolates from 2008 to 2013 [4, 5], and there have been a number of outbreaks reported both in the UK and in Europe [5]. In England, most known CPE transmission occurs in hospitals, and consequently various key guidance documents exist on the management of colonisation or infection due to CPE in England [6] in order to prevent or reduce their spread into (and within) healthcare settings. These advocate a range of precautions including the rapid identification of CPE-colonised patients, isolation (and barrier nursing) to prevent onward transmission, adherence to hand hygiene guidelines, and stringent cleaning and decontamination of patient bed spaces and equipment to ensure that there is no risk to other patients or staff members.

Despite greater awareness of the importance of environmental decontamination for reducing HAI risk, cleaning can often be inefficient especially in busy and understaffed wards [7]. Several authors have investigated novel technologies for whole room decontamination (including ultraviolet (UV) light [8, 9], hydrogen peroxide vapour (HPV) [8], and ozone [10]) that could be used to supplement the standard cleaning protocols for CPEs and other HAIs. These technologies all have broad antimicrobial activity, with UV treatment of rooms spiked with a range of nosocomial pathogens resulting in 2–4 log_10_ reductions in bacterial counts [11]. These technologies have a major disadvantage in that rooms need to be sealed and unoccupied at the time of application.

The blue wavelengths within the visible light spectrum (especially wavelengths between 400 nm to 470 nm) are intrinsically antimicrobial and do not require additional exogenous photosensitizers to exert an antimicrobial effect [12]. Photodynamic inactivation of bacterial cells occurs due to photo-excitation of intracellular porphyrins [13] by the blue light (BL), leading to energy transfer and the production of highly cytotoxic reactive oxygen species (ROS); primarily singlet oxygen (^1^O_2_) [12, 14–16]. Although less germicidal compared to ultra-violet light [13], pathogens can be selectively inactivated whilst preserving human cells and consequently BL is considered much less detrimental to mammalian cells [17, 18].

BL has been shown to exhibit a broad spectrum of antimicrobial effect against bacteria and fungi [19–21], and has been investigated as a new disinfection technology termed the HINS-light environmental decontamination system (HINS-EDS) [13, 22–24]. The HINS-EDS is a type of ceiling-mounted lightbulb which delivers low-irradiance 405 nm light continuously and is suitable for use in patient occupied settings. Evaluation studies showed that there was a statistically significant 90% reduction in numbers of culturable *Staphylococci* spp. following 24 h of use in an unoccupied room [22], and reductions of 56–86% when used in burns isolation rooms occupied by MRSA-positive patients. Furthermore, when the system was no longer used, the room became recontaminated to levels similar to those pre-treatment.

This study was performed to investigate whether 405 nm blue light is effective against CPEs in the in vitro setting. Given that the majority of environmental contamination involves bacteria growing/persisting as biofilms, coupled with the higher tolerance of biofilms to decontamination agents, we investigated the effects of BL on mature biofilms.

## Methods

A series of in vitro experiments were conducted with a panel of organisms (Table 1) to determine the efficacy of blue light (405 nm) against CPE bacteria growing as mature biofilms. The panel comprised 12 well-characterised clinical CPE isolates recovered from clinical samples at the Queen Elizabeth Hospital in Birmingham. All isolates were highly antibiotic resistant and were recovered from a variety of sources. Typing using a discriminatory approach (e.g. variable number tandem repeat (VNTR) for *Klebsiella* spp., and multi-locus sequence typing (MLST) for both *Klebsiella* spp. and *E.coli*) was performed by relevant reference laboratories for all isolates prior to inclusion in the study.

**Table 1:** The characteristics (species, source, resistance mechanism, and antibiogram) of the CPE isolates

Study identifier	Bacterium	Source	Resistance mechanism^a^	Antibiogram													
				AMO	AUG	TAZ	CXM	CEO	CET	CAZ	CPM	AZT	ERT	MER	AMI	CIP	CLX
CPE_8180	*K. pneumoniae*	Groin swab	*bla*_NDM_	R	R	R	R	R	R	R	R	R	R	R	S	R	R
CPE_8770	*K. pneumoniae*	Drain	*bla*_NDM_	R	R	R	R	R	R	R	R	R	R	R	S	R	R
CPE_9606	*E. coli*	Tip of cannula	*bla*_NDM_	R	R	R	R	R	R	R	R	R	R	R	S	R	R
CPE_7534	*E. coli*	Wound	*bla*_NDM_	R	R	R	R	R	R	R	R	R	R	R	S	R	R
CPE_5773	*K. pneumoniae*	Faeces	*bla*_NDM_ & *bla*_OXA-48_	R	R	R	R	R	R	R	R	R	R	R	R	R	R
CPE_6949	*K. pneumoniae*	Urine	*bla*_KPC_	R	R	R	R	R	R	R	I	R	R	R	S	S	R
CPE_0257	*K. pneumoniae*	Urine	*bla*_KPC_	R	R	R	R	R	R	R	R	R	R	R	S	I	R
CPE_4388	*K. oxytoca*	Sputum	*bla*_KPC_	R	R	R	R	R	R	R	I	R	R	R	S	S	R
CPE_1798	*K. pneumoniae*	Urine	*bla*_OXA-48_	R	R	R	R	R	R	R	R	R	R	I	S	R	R
CPE_8421	*E. coli*	Drain	*bla*_OXA-48_	R	nt	R	R	R	R	R	R	R	R	R	S	S	nt
CPE_9956	*K. pneumoniae*	Urine	*bla*_NDM_ and *bla*_OXA-48_	R	R	R	R	R	R	R	R	R	R	R	R	R	R
CPE_7855	*K. pneumoniae*	Urine	*bla*_NDM_ and *bla*_OXA-48_	R	R	R	R	R	R	R	R	R	R	R	S	R	R

Notes: ^a^ as determined through PCR testing at reference laboratory Antibiogram: R (resistant), I (intermediate), S (sensitive), nt (not tested). AMO (Amoxicillin), AUG (Augmentin), TAZ (Piperacillin tazobactam), CXM (Cefuroxime), CEO (Cefoxitin), CET (Cefotaxime), CAZ (Ceftazidime), CPM (Cefepime), AZT (Aztreonam), ERT (Ertapenem), MER (Meropenem), AMI (Amikacin), CIP (Ciprofloxacin), CLX (Cephalexin)

All isolates were stored at − 80 °C on Protect™ beads, and were routinely cultured on cysteine lactose electrolyte deficient (CLED) or blood agar prior to each experiment. Experiments were designed to assess the antibacterial activity of blue light against biofilm growth forms of the panel of bacteria described above.

### Blue light equipment

High intensity blue light was provided by a LED (light emitting diode) Flood array (Henkel-Loctite, Hemel Hempstead, UK). This array utilises 144 reflectorized LEDs which produce a homogeneous illuminated area of 10 cm × 10 cm. The emission spectrum of the LED array was determined using a USB2000 spectrophotometer (Ocean Optics, Oxford, UK), and the platform calibrated to deliver a reproducible irradiance of 60 mW/cm^2^ when the LED array was positioned 15.5 cm above the test area.

### Impact of blue light on pre-formed biofilms

**Fig. 1:**
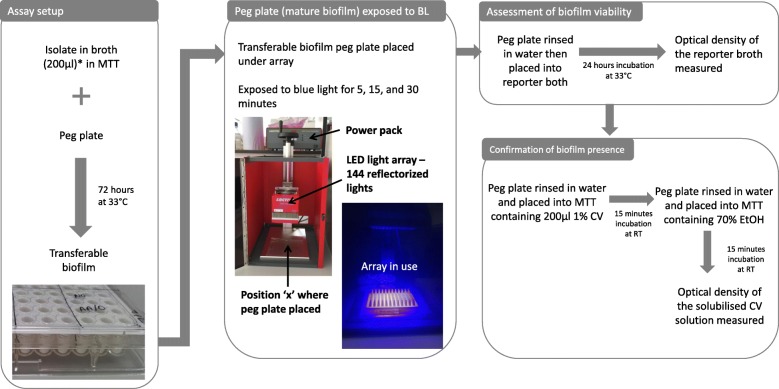
Flow diagram showing the stages of the MBEC assay

The antibacterial activity of blue light against pre-formed biofilms was assessed by conducting ‘minimum biofilm eradication concentration’ (MBEC) experiments [25] on each isolate (Fig. 1). Overnight LB cultures of the test strains (made by inoculating approximately three to five colonies into 5 ml of fresh Luria Broth (LB) broth and incubating at 37 °C overnight) were diluted in fresh antibiotic-free Mueller-Hinton (MH) broth to an optical density (OD) at 600 nm of 0.1 and then 200 μl seeded into wells of a 96-well microtiter tray (MTT). Positive (200 μl 0.1 OD_600_ diluted organisms) and negative (200 μl MH broth) controls were included per blue light time point to be tested.

To produce a ‘transferable biofilm’, a 96 well polypropylene plate [Starlabs, UK] was then placed into the MTT so that each well contained a ‘peg’, on which biofilms could form, before the plates were sealed, and statically incubated at 33 °C for 72 h. After 72 h, the pegs (±biofilm) were removed and washed in a MTT containing sterile water (to remove any unbound cells). The positive and negative control ‘peg plate’ was placed in a clean, empty MTT and wrapped in foil. Following this, both the control and the test peg plate were placed in the test area (15.5.cm beneath the light source) and exposed to the blue light for time points of 15 or 30 min (corresponding to a blue light dose of 54 or 108 J/cm^2^ respectively). Shorter BL exposures were also performed by reducing the distance between the array and the light source, so that a 5 min exposure delivered the same dose of BL as a 45 min exposure (162 J/cm^2^).

The foil around the control plate prevented the pegs from receiving any blue light treatment (and hence these positive control biofilms were not exposed to the blue light), but the control plate biofilms would have most likely been exposed to the same amount of heating and drying as the blue light exposed test plate.

After the treatment, the peg plates were carefully placed into a MTT containing 200 μl sterile MH broth (herein referred to as ‘reporter broth’) for overnight incubation. After 18-24 h, the OD of the reporter broth was measured to assess the viability (seeding) of the biofilms following blue light exposure.

To demonstrate the presence of biofilms on the pegs, crystal violet (CV) assays were additionally performed on the pegs after the OD of the reporter broth had been measured. This involved placing the pegs into MTTs containing 200 μl of 1% CV (which binds to any present microbial biomass of biofilm), followed by washing (to remove unbound CV) and subsequent solubilisation of the CV in 200 μl of 70% ethanol. The peg biofilm biomass could then be measured using OD readings as previously and the presence of the biofilm confirmed. Two biological and 10 technical replicates were performed for each strain and blue light exposure duration, respectively.

The blue light dose (J/cm^2^) received by the bacteria was calculated by multiplying the irradiance of light (W/cm^2^) to which the sample was exposed, by the exposure time (seconds).

### Statistical analysis

The ability of biofilms to seed new growth following exposure to blue light was assessed by comparing the OD values at each blue light time point (5, 15, and 30 min) versus the untreated (positive) control. An initial check of the data using ggplot2 [26] demonstrated that the data were non-normal in distribution (data not shown), and consequently the non-parametric Mann-Whitney U test was used to determine significance (*p* < 0.05) and produce estimates of the median of the difference between sets of OD values, along with 95% confidence intervals. The software package R [27] (version 3.4.3) was used for all analysis.

## Results

All isolates were confirmed to be genetically diverse and unrelated via typing (VNTR and MLST) (Additional file 1: Tables S1, S2 and Additional file 2: Figure S1 and Additional file 3: Figure S2), and hence were included in the study. All were susceptible to BL treatment, with statistically significant (*p* < 0.05) reductions in biofilm seeding (of 72 h biofilms) observed when compared to an untreated positive control. There was no significant difference in the seeding of the positive controls across all time points (data not shown), and all isolates (apart from CPE_8180) were similarly susceptible. In contrast to previously published data [28], there were no isolates where BL treatment resulted in increased biofilm seeding.

**Fig. 2:**
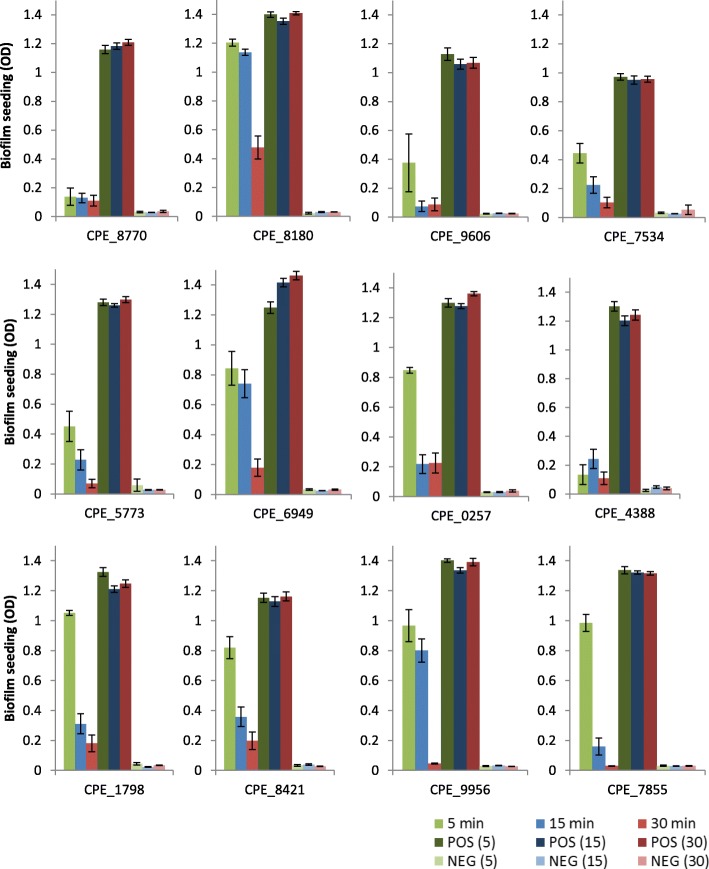
Showing the mean average biofilm seeding for all isolates following testing with blue light

A time-dependent effect was noticed, with longer exposure times resulting in greater reductions in seeding (Fig. 2). For example, at five minutes exposure, ≥80% reductions in seeding were observed for two isolates (CPE_5773 and CPE_9606), compared to five isolates at 15 min (CPE_5773, CPE_7855, CPE_0257, CPE_8770 and CPE_9606), and 11 isolates at 30 min (all except for CPE_8180) (Table 2).

**Table 2:** The percentage reductions in biofilm seeding following testing with the three doses of BL

Study identifier	Bacterium	Resistance mechanism^	Percentage (%) reductions in biofilm seeding following BL exposure								
			5 min (162 J/cm^2^)			15 min (54 J/cm^2^)			30 min (108 J/cm^2^)		
			% reduction	n	*p*-value	% reduction	n	p-value	% reduction	n	*p*-value
CPE_8180	*K. pneumoniae*	*bla*_NDM_	13.9	20	< 0.0001	15.9	40	< 0.0001	66.1	40	< 0.0001
CPE_8770	*K. pneumoniae*	*bla*_NDM_	**88.1**	20	< 0.0001	**89.0**	40	< 0.0001	**90.9**	40	< 0.0001
CPE_9606	*E. coli*	*bla*_NDM_	66.7	20	< 0.0001	**93.0**	40	< 0.0001	**91.8**	40	< 0.0001
CPE_7534	*E. coli*	*bla*_NDM_	54.2	40	< 0.0001	76.3	40	< 0.0001	**89.1**	40	< 0.0001
CPE_5773	*K. pneumoniae*	*bla*_NDM_ & *bla*_OXA-48_	64.7	20	< 0.0001	**81.9**	40	< 0.0001	**94.6**	40	< 0.0001
CPE_6949	*K. pneumoniae*	*bla*_KPC_	32.5	20	0.0023	47.7	40	< 0.0001	**87.7**	40	< 0.0001
CPE_0257	*K. pneumoniae*	*bla*_KPC_	34.8	20	< 0.0001	**82.9**	40	< 0.0001	**83.4**	40	< 0.0001
CPE_4388	*K. oxytoca*	*bla*_KPC_	**89.8**	20	< 0.0001	79.8	40	< 0.0001	**91.2**	40	< 0.0001
CPE_1798	*K. pneumoniae*	*bla*_OXA-48_	20.6	20	0.0202	74.3	40	< 0.0001	**85.5**	40	< 0.0001
CPE_8421	*E. coli*	*bla*_OXA-48_	28.9	40	0.0044	68.3	40	< 0.0001	**82.9**	40	< 0.0001
CPE_9956	*K. pneumoniae*	*bla*_NDM_ & *bla*_OXA-48_	31.0	20	< 0.0001	40.0	40	< 0.0001	**96.7**	40	< 0.0001
CPE_7855	*K. pneumoniae*	*bla*_NDM_ & *bla*_OXA-48_	26.3	20	0.0015	**87.9**	40	< 0.0001	**97.7**	40	< 0.0001

Notes: Resistance mechanism: ^ as determined through PCR testing at reference laboratory. *bla*_NDM_ (gene encoding for New Delhi metallo-beta-lactamase 1), *bla*_KPC_ (gene encoding for *Klebsiella pneumoniae* carbapenemase), *bla*_OXA-48_ (gene encoding oxacillinase OXA-48)

Bold numbers represent results where there was ≥80% reductions in biofilm seeding

‘n’ refers to the number of technical replicates that were performed

Interestingly there was not a dose-dependent effect of BL. Although the dose of BL was modified so that it was higher at 5 min (162 J/cm2) than at 15 or 30 mins (doses of 54 J/cm2 and 108 J/cm2, respectively), this is not reflected in the results, with the smallest reductions in seeding observed at five minutes for the majority of isolates (11/12) (Table 2). At this dosage, reductions in seeding ranged from 13.9% (CPE_8180) to 89.8% (CPE_4388).

BL treatment of the mature CPE biofilms also had anti-biofilm effects, with reductions in biofilm biomass observed for all isolates when the CV data for the exposed biofilm was compared to the unexposed positive control (Additional file 4: Figure S3).

## Discussion

Environmental contamination of a range of hospital surfaces is recognised as a source of patient infection, and improved cleaning and disinfection methods are continually being sought. Various studies [22–24] have shown the effectiveness of a ceiling-mounted 405 nm light system (the HINS-light EDS) for continuous decontamination of the air and all exposed surfaces within the environment. This system emits 405 nm BL at an irradiance of 0.1–0.5 mW/cm2 [13], and has undergone numerous clinical evaluations within in-patient and out-patient settings as mentioned previously. However, to our knowledge, no studies exist which examine the effectiveness of this wavelength of BL against multidrug resistant organisms, including CPEs.

We have shown in this small study that 405 nm BL is universally effective against a small panel of genetically diverse CPEs, achieving equal reductions in seeding of mature biofilms, regardless of antibiotic susceptibility or antibiotic resistance mechanisms carried by the specific isolates. All results were statistically significant (*p* < 0.05), and in contrast to our previous work [28], there were no increases in biofilm seeding in response to BL (which we previously hypothesised was the result of sub-lethal BL stress of the isolates concerned). We also found concurrent reductions in biofilm biomass, demonstrating that BL has anti-biofilm effects, as well as effectively reducing seeding.

There is evidence of reduced susceptibility in one *K. pneumoniae bla*_NDM_ CPE isolate (CPE_8180), with modest reductions in seeding (of 66.1%) after 30 min BL exposure. There were reductions of 90.9% with a similar *K. pneumoniae bla*_NDM_ CPE isolate (CPE_8770) after the same exposure time and dose, and this difference cannot be explained by antibiogram since both isolates had identical susceptibilities (Table 1). Porphyrins are reported to be key for the mechanism of action (MOA) of BL [14, 20], however recent work has demonstrated that viruses (that lack porphyrins) are also susceptible [29]. Feline calicivirus (FCV) was used as a surrogate for norovirus, and exposed to 405 nm BL whilst suspended in minimal, and organically-rich media. A plaque assay performed to assess how the virus infectivity changed following BL exposure, revealed a 4 log_10_ (99.99%) reduction in infectivity of FCV in minimal media after a dose of 2.8 KJ/cm^2^ [29, 30]. Interestingly, there was enhanced inactivation when FCV was present in the organically-rich media [29]. The molecular basis of BL susceptibility would be an interesting avenue to investigate further, both to ascertain why CPE_8180 had lower susceptibility, and to further elucidate the MOA.

There are various limitations of this work. Owing to time constraints, we only tested a small panel of 12 CPE producers, and these were restricted to *bla*_NDM,_ *bla*_OXA-48_ and *bla*_KPC,_ produced by *Klebsiella pneumoniae, K. oxytoca* and *E. coli.* To make this work more applicable, it would be useful to additionally test isolates producing *bla*_VIM_ and *bla*_IMP_, as these are also important carbapenemases globally. Although we have tested BL against a number of *A. baumannii* previously [28], we could also have included pan-resistant species in this current study (such as *bla*_OXA_ positive *A. baumannii*), and acknowledge this as a limitation.

For simplicity, all experiments were performed on monomicrobial biofilms growing on a polystyrene plastic surface. To ensure that results are applicable to the clinical setting, experiments should be performed on a range of materials present in the clinical setting (e.g. metals, wood, plastics, painted surfaces), to account for any differences in biofilm formation or absorption of BL. Also, it would be prudent to investigate whether BL remains effective when biofilms contain a variety of species of bacteria and/or viruses. Also, to conform to standard tests of decontamination, we would need to measure what a 90% reduction (for example) means in terms of log_10_ reductions, and perform tests comparing this technology to conventional cleaning measures to ensure non-inferiority of BL, and see whether there is an additive effect.

In terms of translating this technology, the BL array would need to be incorporated into a suitable delivery platform, either a light fitting or ‘robot’ (for decontamination of a room or clinical setting) or a hand held device/wand. The latter would be of most use for the decontamination of small areas, but could also be explored further for superficial wound decontamination/ treatment. This would require collaboration with industrial partners and extensive additional testing. The potential for bacteria to develop resistance to BL would also need to be investigated once a prototype platform has been developed.

It is hypothesised that tolerance is unlikely owing to the non-selective nature of the MOA and resulting ROS-damage [31], but there were conflicting results from a number of studies [32, 33]. Tomb et al. [30, 34] repeatedly exposed MSSA and MRSA isolates to sub-lethal doses of BL (108 J/cm^2^ chosen since it resulted in 98% inactivation in previous studies) for 15 cycles, and enumerated surviving colonies after each cycle. These counts were assessed over the 15 cycles and no statistically significant differences were identified, suggesting that sub-lethal BL doses do not promote bacterial tolerance. This further supports BL as a promising new agent for clinical decontamination.

## Conclusions

We have shown in this small study that BL is effective at reducing the seeding of mature CPE biofilms in vitro, and offers great promise as a topical decontamination/treatment agent for both clinical and environmental applications. Further research is warranted to investigate activity on polymicrobial biofilms existing on a range of other clinically relevant materials and to develop a suitable prototype for clinical translation of this promising technology.

## Additional files

Additional file 1: **Table S1:** Showing the VNTR profiles and MLST types (where available) for the *Klebsiella pneumoniae* and *K. oxytoca* isolates used in the study. **Table S2:** Showing the MLST types (where available) for the *Escherichia coli* isolates used in the study. (DOCX 17 kb)

Additional file 2: **Figure S1:** Core phylogeny tree showing the relatedness of the seven *Klebsiella pneumoniae* isolates that were included in the study. (PDF 278 kb)

Additional file 3: **Figure S2:** Core phylogeny tree showing the relatedness of the three *Escherichia coli* isolates that were included in the study. (PDF 254 kb)

Additional file 4: **Figure S3:** showing the mean average biofilm biomass for all isolates following testing with blue light. (PDF 182 kb)

## Abbreviations

BL: Blue light
CLED: Cysteine lactose electrolyte deficient
CPE: Carbapenemase-producing *Enterobacteriaceae*
CV: Crystal violet
FCV: Feline calicivirus
HINS-EDS: HINS-light environmental decontamination system
HPV: Hydrogen peroxide vapour
LB: Luria Broth
LED: Light emitting diode
MBEC: Minimum biofilm eradication concentration
MH: Mueller-Hinton broth
MOA: Mechanism of action
MTT: 96-well microtiter tray
OD: Optical density
ROS: Reactive oxygen species
UV: Ultraviolet light
