# Supplementary material for: The potential of visible blue light (405 nm) as a novel decontamination strategy for carbapenemase-producing enterobacteriaceae (CPE)
Source: Antimicrob Resist Infect Control. 2019 Jan 17;8:14. doi: 10.1186/s13756-019-0470-1 (PMC6335786; doi:10.1186/s13756-019-0470-1)
Supplement: Supplementary file 1 — Table S1: Showing the VNTR profiles and MLST types (where available) for the Klebsiella pneumoniae and K. oxytoca isolates used in the study. Table S2: Showing the MLST types (where available) for the Escherichia coli isolates used in the study. (DOCX 17 kb) [file 13756_2019_470_MOESM1_ESM.docx]

Additional file 1: Table S1: Showing the VNTR profiles and MLST types (where available) for the *Klebsiella pneumoniae* and *K. oxytoca* isolates used in the study

| Study identifier | Bacterium | VNTR profile | MLST type |
| --- | --- | --- | --- |
| CPE_8180 | *K. pneumoniae* | 5,4,1,1,-,2,4,4,3,2,3 | Unassigned^ |
| CPE_8770 | *K. pneumoniae* | 7,3,1,5,1,2,4,1,4,2,4 | 29 |
| CPE_5773 | *K. pneumoniae* | 5,4,1,1,-,2,4,4,3,2,3 | 101* |
| CPE_6949 | *K. pneumoniae* | Not typed | 25 |
| CPE_0257 | *K. pneumoniae* | 5,2,3,15,-,2,3,3,3,3,4 | 1373 |
| CPE_4388 | *K. oxytoca* | Not typed | Unassigned |
| CPE_1798 | *K. pneumoniae* | Not typed | 16 |
| CPE_9956 | *K. pneumoniae* | 6,3,4,0,1,1,4,1,5,2,3 | 14 |
| CPE_7855 | *K. pneumoniae* | 5,4,1,1,-,2,4,4,3,2,3 | 101 |

Notes:

* There are 18 SNPs different between CPE_5773 and CPE_7855.

^ There are 28,738 SNPs different between CPE_8180 and CPE_5773

Additional file 1: Table S2: Showing the MLST types (where available) for the *Escherichia coli* isolates used in the study

| Study identifier | Bacterium | MLST type |
| --- | --- | --- |
| CPE_9606 | *E. coli* | 1702 |
| CPE_7534 | *E. coli* | 1284 |
| CPE_8421 | *E. coli* | 38 |
