# Supplementary material for: The potential of visible blue light (405 nm) as a novel decontamination strategy for carbapenemase-producing enterobacteriaceae (CPE)
Source: Antimicrob Resist Infect Control. 2019 Jan 17;8:14. doi: 10.1186/s13756-019-0470-1 (PMC6335786; doi:10.1186/s13756-019-0470-1)
Supplement: Supplementary file 3 — Figure S2: Core phylogeny tree showing the relatedness of the three Escherichia coli isolates that were included in the study. (PDF 254 kb) [file 13756_2019_470_MOESM3_ESM.pdf]

Supplementary Figure 2: Core phylogeny tree showing the relatedness of the three *Escherichia coli* isolates that were included in the study.

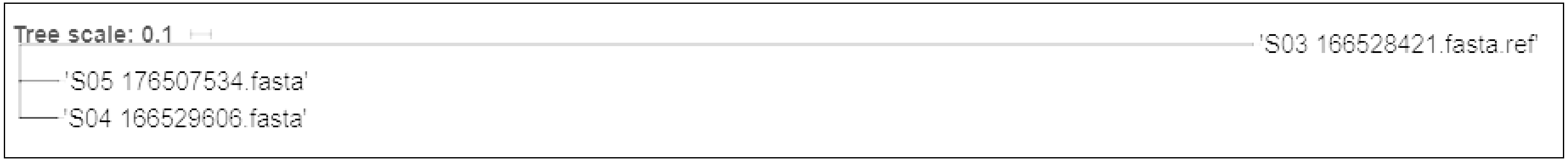

Notes:
The last four digits of the specimen names on the tree correspond to the last 4 in the isolate identifier. E.g. S03 166528421 is CPE_8421
The scale used for the branch lengths is shown in the top left of the figure
