# Supplementary material for: The potential of visible blue light (405 nm) as a novel decontamination strategy for carbapenemase-producing enterobacteriaceae (CPE)
Source: Antimicrob Resist Infect Control. 2019 Jan 17;8:14. doi: 10.1186/s13756-019-0470-1 (PMC6335786; doi:10.1186/s13756-019-0470-1)
Supplement: Supplementary file 4 — Figure S3: showing the mean average biofilm biomass for all isolates following testing with blue light. (PDF 182 kb) [file 13756_2019_470_MOESM4_ESM.pdf]

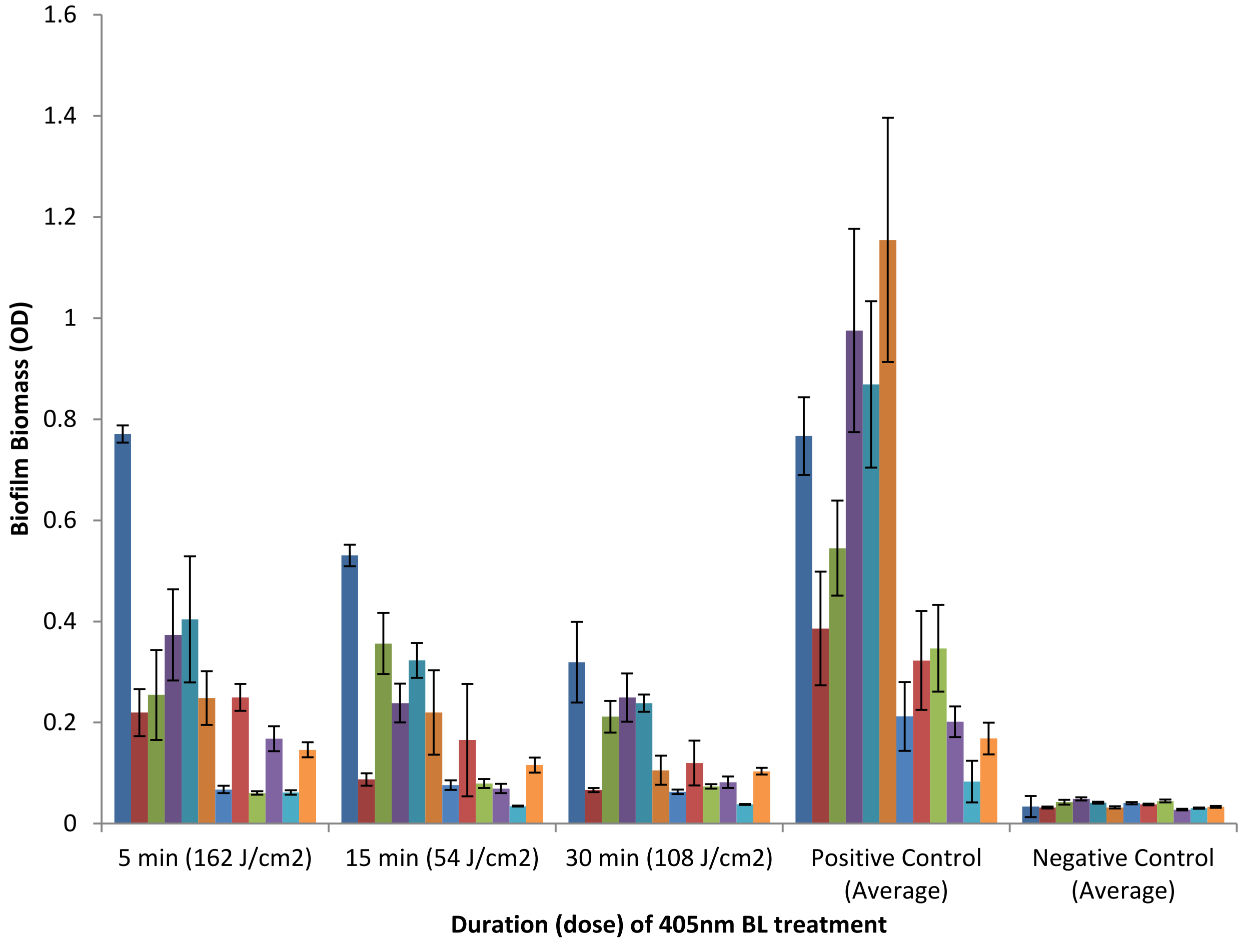

Suppl Figure 3: Graph showing the mean average biofilm biomass results for all isolates when they were tested with blue light (BL) for 5, 15, and 30 minutes.

Biofilm biomass (OD) on the y-axis refers to the mean average biofilm biomass for the isolates tested after exposure to blue light at the range of durations tested (in minutes) on the x-axis.

Each data bar represents the mean average biofilm biomass (crystal violet values) from 20-40 technical replicates (as detailed on Table I). The standard error of the mean is also plotted. The positive control represents the average biofilm biomass per isolate in the absence of BL treatment across all timepoints. The negative control represents the background level of optical density for the reagents used in the assay.
